# Antiobesity and lipid-lowering effects of *Bifidobacterium *spp. in high fat diet-induced obese rats

**DOI:** 10.1186/1476-511X-10-116

**Published:** 2011-07-12

**Authors:** Hyang Mi An, Shin Young Park, Do Kyung Lee, Jung Rae Kim, Min Kyeong Cha, Si Won Lee, Hyung Taeck Lim, Kyung Jae Kim, Nam Joo Ha

**Affiliations:** 1 College of Pharmacy, Sahmyook University, Seoul 139-742, Republic of Korea; 2 Jahayun oriental medicine clinic, Seoul 135-897, Republic of Korea

## Abstract

**Background:**

Recent studies have reported the preventive effects of probiotics on obesity. Among commensal bacteria, bifidobacteria is one of the most numerous probiotics in the mammalian gut and are a type of lactic acid bacteria. The aim of this study was to assess the antiobesity and lipid-lowering effects of *Bifidobacterium *spp. isolated from healthy Korean on high fat diet-induced obese rats.

**Methods:**

Thirty-six male Sprague-Dawley rats were divided into three groups as follows: (1) SD group, fed standard diet; (2) HFD group, fed high fat diet; and (3) HFD-LAB group, fed high fat diet supplemented with LAB supplement (*B. pseudocatenulatum *SPM 1204, *B. longum *SPM 1205, and *B. longum *SPM 1207; 10^8 ^~ 10^9 ^CFU). After 7 weeks, the body, organ, and fat weights, food intake, blood serum levels, fecal LAB counts, and harmful enzyme activities were measured.

**Results:**

Administration of LAB reduced body and fat weights, blood serum levels (TC, HDL-C, LDL-C, triglyceride, glucose, leptin, AST, ALT, and lipase levels), and harmful enzyme activities (β-glucosidase, β-glucuronidase, and tryptophanase), and significantly increased fecal LAB counts.

**Conclusion:**

These data suggest that *Bifidobacterium *spp. used in this study may have beneficial antiobesity effects.

## Background

Obesity, a condition in which an abnormally large amount of fat is stored in the adipose tissue, resulting in an increase in body weight, is one of the major public health problems in the United States and other developed countries. In general, it is accepted that obesity results from disequilibrium between energy intake and expenditure [1], and this condition has a great impact on several metabolic and chronic ailments including heart disease, cancer, arthritis, obstructive sleep apnea, hypertension, hyperlipidemia, and type 2 diabetes associated with insulin resistance [2]. To date, pharmacological treatments do not appear to be effective in producing sustained long-term weight loss [3]. Therefore, further research is needed to discover new drug therapies that can be used to reduce the prevalence of obesity.

Probiotics are defined as viable microbial dietary supplements that exert beneficial effects on host health [4]. Probiotics have attracted public attention because of their potential effectiveness for both the prevention and the treatment of immune diseases [5]. In addition, recent experimental studies have demonstrated the preventive effects of some bacterial strains on obesity. Among commensal bacteria, bifidobacteria are one of the most numerous probiotics in the mammalian gut and are a type of lactic acid bacteria. Bifidobacteria are widely used and well tolerated. In one study, a strain of *Bifidobacterium longum *exhibited a more significant effect in lowering serum total cholesterol than a mixed culture of *Streptococcus thermophilus *and *Lactobacillus delbrueckii *subspecies *bulgaricus *(SL) both in rats and humans [6]. Another study found that in probiotic treated-mice, *Bifidobacterium *spp. significantly and positively correlated with improved glucose-tolerance, glucose-induced insulin-secretion, and normalized inflammatory tone (decreased endotoxemia, plasma and adipose tissue pro-inflammatory cytokine) [7]. Finally, VSL no. 3, a mixture of viable lyophilized bifidobacteria, lactobacilli and *Streptococcus thermophilus*, improved diet-induced obesity and its related hepatic steatosis and insulin resistance by increasing hepatic natural killer T-cells and reducing inflammatory signaling in mice [8]. These data suggest that some specific strains of bifidobacteria related to lipid metabolism and body weight may be potential therapeutic candidates for management of obesity.

In the present study, we used a LAB supplement of *Bifidobacterium pseudocatenulatum *SPM 1204 (immuno-enhancement and anti-microbial effects) [9], *Bifidobacterium longum *SPM 1205 (inhibitory effect on harmful enzyme activities of intestinal microflora) [10], and *Bifidobacterium longum *SPM 1207 (hypocholesteremia effect) [11], and investigated the antiobesity and lipid-lowering effects of *Bifidobacterium *spp. on high fat diet-induced obese rats.

## Materials and methods

### Bacterial strains used in this study

The culture media and methods used in this study for the isolation and identification of bacterial strains are shown in Table 1. For isolation of *B. pseudocatenulatum *SPM 1204, *B. longum *SPM 1205, and *B. longum *SPM 1207 used in this study, fecal samples from healthy Koreans (20-30 years old) were collected by BBL's anaerobic sample collection and transport system to maintain anaerobic conditions, and were used within 24 h. Fecal samples were serially diluted 10-fold from 10^-1 ^to 10^-8^, and 0.1 ml was spread onto selective Blood Liver (BL) agar medium containing 5% sheep blood. After 48-h incubation in anaerobic conditions at 37°C, brown or reddish-brown colonies 2-3 mm in diameter were selected for further identification [12]. A fructose-6-phosphate phosphoketolase (F6PPK) test was performed [13] to ensure that the colonies selected were bifidobacteria. To identify the isolated *Bifidobacterium *spp. at the species level, 16S rRNA sequencing was performed by Bioleaders (Daejeon, Korea).

**Table 1:** Culture media and methods

Media	Main enumerated microorganisms	Incubation time (day)
Anaerobic culture^a^		
BL agar medium^b^	Predominant anaerobes	3
GAM agar medium^c^	*Bifidobacterium *spp.	1-2

^a^Culture media were prepared and dispensed into Petri dishes on a clean bench. Dishes were stored in a bactron anaerobic chamber (Sheldon Manufacturing Inc., USA) containing 90% N_2_, 5% CO_2 _and 5% H_2 _until plating. A sample of each dilution was plated on each medium in an anaerobic glove box. Plated dishes were incubated in a bactron anaerobic chamber filled with CO_2 _gas. The presence of O_2 _was monitored with steel wool soaked in acidified copper sulfate solution.

^b^BL, Blood Liver (Nissui Pharm. Co. Ltd., Japan).

^c^GAM, General Anaerobic Medium (Nissui Pharm. Co. Ltd., Japan).

### Animals and treatment

A total of 36 male Sprague-Dawley (SD) rats were purchased from Central Lab Animal Inc. (Korea) at three weeks of age and fed a standard diet (AIN-76A #100000, Dyets Inc., Bethlehem, PA, USA) for 1 week to stabilize all metabolic conditions. Food and water were supplied ad libitum. Each cage contained two rats. The rats were exposed to a 12-h light/dark cycle and maintained at a constant temperature of 32 ± 2°C and humidity of 55 ± 5%. All animal experimentation was performed according to the guidelines for the care and use of laboratory animals approved by Sahmyook University. The rats were randomly selected and assigned to three groups (12 rats per group) according to the type of diet and test-material. As shown in Table 2, Group 1 SD was fed a standard diet and PBS (as control); Group 2 HFD was fed a high fat diet (40% beef tallow modified AIN-76A purified rodent diet #101556, Dyets Inc., Bethlehem, PA, USA) and PBS; and Group 3 HFD-LAB was fed a high fat diet and LAB (*B. pseudocatenulatum *SPM 1204, *B. longum *SPM 1205, and *B. longum *SPM 1207; 1:1:1 ratio) supplement. The composition of the experimental diet is shown in Table 3. All rats were acclimatized to the respective diets for a week before the experiment started. Then the rats were orally administered 0.2 ml of PBS or LAB (10^8 ^~ 10^9 ^CFU) once a day for 5 weeks. Their body weight and food intake were measured daily.

**Table 2:** Feeding schedules used in the present study

Groups	Rats (n)	Food	Treatment (oral administration for 5 weeks)
SD	12	Standard Diet	0.2 ml of sterilized PBS
HFD	12	40% High Fat Diet	0.2 ml of sterilized PBS
HFD-LAB	12	40% High Fat Diet	0.2 ml of LAB (10^8 ^~10^9 ^CFU) in sterilized PBS

**Table 3:** Compositions of experimental diet

	Standard Diet		High Fat Diet	
Ingredients	(AIN-76A diet #100000)		(AIN-76A diet #101556)	
	
	g	kcal	g	kcal
Casein	200	720	200	720
Beef tallow	0	0	400	3600
Methionine	3	12	3	12
Starch	150	540	150	540
Sucrose	500	2000	150	600
Cellulose	50	0	50	0
Corn oil	50	450	0	0
Salt mixture	35	30.8	35	30.8
Vitamin mixture	10	39	10	39
Choline bitartrate	2	0	2	0

Total	1000	3791.8	1000	5541.8

### Analysis of blood serum and organ weight

At the end of the 7-week experimental period, blood samples from each rat were collected into tubes using cardiac puncture. The serum was separated from the blood by centrifugation at 3,500 rpm for 10 min. The total cholesterol (TC), high density lipoprotein cholesterol (HDL-C), low density lipoprotein cholesterol (LDL-C), triglyceride, glucose, leptin, alanine aminotransferase (AST), aspartate aminotransferase (ALT) levels in the serum were analyzed by Korea Animal Medical Science Institute (Korea). Also, the fat (epididymal and retroperitoneal), liver, spleen and kidney were removed immediately after the sacrifice and weighed.

### Measurement of α-amylase and lipase activities

The activities of α-amylase and lipase were measured with the α-Amylase Assay Kit (BioAssay Systems, USA) and Lipase Assay Kit (BioAssay Systems, USA), respectively.

### Fecal sampling and bacteriological analysis

Fecal samples were collected from the rats weekly to determine the total LAB counts and harmful enzyme activities. Fecal samples were taken directly from the rectum by rectal stimulation and immediately transferred into sterile tubes and stored at 4°C.

Fecal samples (0.1 g) were suspended in 0.9 ml of 0.1 M phosphate buffer (pH 6.8 containing 0.5% cysteine) by vortexing, and 0.1 ml was then serially diluted 10-fold from 10^-1 ^to 10^-7^. 1 ml was then poured into selective General Anaerobic Medium (GAM) agar medium (Table 1). After 48 h of incubation under anaerobic conditions, colonies were counted as *Bifidobacterium *spp. [10]. The numbers of colony forming units (CFU) are expressed as log_10 _CFU per gram.

### Harmful enzyme activities of intestinal microflora

The harmful activities of enzymes such as β-glucosidase, β-glucuronidase, tryptophanase, and urease of intestinal microflora related to colon cancer were tested in fecal samples of rats as previously described [14-16].

### Assay of β-glucosidase activity

β-glucosidase activity was assayed in a 2-ml reaction mixture containing 0.8 ml of 2 mM p-nitrophenyl-β-D-glucopyranoside and 0.2 ml of enzyme solution (suspended fecal sample). The reaction was incubated for 30 min at 37°C and then stopped by adding 1 ml of 0.5 N NaOH. The reaction mixture was then centrifuged at 3,000 rpm for 10 min. Enzyme activity was measured by monitoring absorbance at 405 nm.

### Assay of β-glucuronidase activity

β-glucuronidase activity was assayed in a 2-ml reaction mixture consisting of 0.8 ml of 2 mM p-nitrophenyl-β-D-glucuronide and 0.2 ml of the enzyme solution. The reaction was incubated for 30 min at 37°C and then stopped by adding 1 ml of 0.5 N NaOH. The reaction mixture was centrifuged at 3,000 rpm for 10 min. Enzyme activity was measured by monitoring absorbance at 405 nm.

### Assay of tryptophanase activity

Tryptophanase activity was assayed in a 2.5-ml reaction mixture consisting of 0.2 ml of complete reagent solution (2.75 mg of pyridoxal phosphate, 19.6 mg of disodium EDTA dihydrate, and 10 mg of bovine serum albumin in 100 ml of 0.05 M potassium phosphate buffer, pH 7.5), 0.2 ml of 20 mM tryptophan, and 0.1 ml of the enzyme solution. The reaction was incubated for 1 h at 37°C and then stopped by adding 2 ml of color reagent solution (14.7 g p-dimethylaminobenzaldehyde in 52 ml H_2_SO_4 _and 948 ml 95% ethanol). The reaction mixture was then centrifuged at 3,000 rpm for 10 min. Enzyme activity was measured by monitoring absorbance at 550 nm.

### Assay of urease activity

Urease activity was assayed in a 0.5-ml reaction mixture consisting of 0.3 ml of urea substrate solution (4 mM urea in 20 mM sodium phosphate buffer, pH 7.0) and 0.1 ml of the enzyme solution. The reaction was incubated for 30 min at 37°C and then stopped by adding 0.1 ml of 1 N (NH_4_)_2_SO_4_. Phenolnitroprusside reagent (1 ml) and alkaline hypochlorite reagent (NaClO, 1 ml) were added to the stopped reaction mixture and incubated for 20 min at 65°C. The reaction mixture was centrifuged at 3,000 rpm for 10 min. Enzyme activity was measured by monitoring absorbance at 603 nm.

### Statistical analysis

Results were expressed as mean ± standard deviation. Significant differences among groups were determined using Duncan's Multiple Range Test (SAS ver. 8.1, SAS Institute Inc., Cary, NC, USA). Values of *P *< 0.05 were considered significant.

## Results

### Body weight, organ weight, fat weight, and food intake

**Figure 1:**
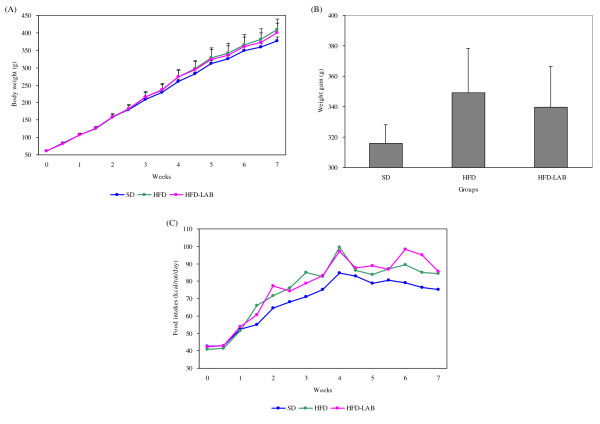
**Effects of LAB during experimental periods**. (A) Body weight, (B) Weight gain, and (C) Food intake. Data are presented as means and standard deviation.

The body weight of all groups was increased every week, especially that of the HFD fed groups compared to that of the SD group. The high fat diet promoted a significant increase in body weight over the seven-week period (Figure 1A). Differences in body weight among groups became noticeable at the fourth week of the diet and became significant after the seventh week. In the HFD-LAB group, significant differences occurred after seven weeks of age. At the end of the experiment, the gain in body weight was higher in the HFD group than in the HFD-LAB group (Figure 1B). The mean body weight increased by 315.86 ± 12.42 g in the SD group, 349.14 ± 29.14 g in the HFD group, and 339.70 ± 26.75 g in the HFD-LAB group. The average food intake of the HFD-fed groups was higher than that of the SD group, but did not differ between the HFD and HFD-LAB groups (Figure 1C). This indicates that the HFD-fed groups had similar caloric consumption. This is a clear sign that the high fat diet induced a profound accumulation of energy in the form of body fat, including epididymal and retroperitoneal fat masses (Table 4). In addition, spleen, kidney, and liver were weighed, but no significant differences were observed.

**Table 4:** Weights of body, fat, spleen, kidney, and liver

	SD	HFD	HFD-LAB
Body weight (g)	381.00 ± 11.95^B^	413.86 ± 30.28^A^	402.20 ± 29.23^AB^
Fat weight (g) (epididymal + retroperitoneal)	13.94 ± 2.66^B^	20.94 ± 4.08^A^	18.47 ± 2.02^A^
Fat pad weight (g/100 g body weight)	3.63 ± 0.65^B^	5.11 ± 0.60^A^	4.83 ± 0.40^A^
Spleen (g)	0.66 ± 0.03	0.60 ± 0.07	0.64 ± 0.10
Kidney (g)	2.59 ± 0.15	2.60 ± 0.18	2.61 ± 0.08
Liver (g)	18.47 ± 1.95^AB^	20.04 ± 2.27^A^	17.59 ± 2.33^B^

Each value provided is the mean ± standard deviation. Values of each group with the same letters for each serological factor were not significantly different (*P *< 0.05).

### Analysis of blood serum levels

The TC, HDL-C, LDL-C, triglyceride, glucose, leptin, AST, ALT, α-amylase and lipase levels in serum in each group are summarized in Tables 5 and 6. Compared with the HFD group, the HFD-LAB group had slightly decreased TC, HDL-C, LDL-C, triglyceride, glucose, leptin, AST, ALT, and lipase levels. α-Amylase levels were not different.

**Table 5:** Serum levels of cholesterol, triglyceride, glucose, leptin, AST, and ALT

	SD	HFD	HFD-LAB
TC (mg/dl)	88.20 ± 12.24	92.20 ± 11.02	89.70 ± 17.33
HDL-C (mg/dl)	54.40 ± 9.25^A^	47.40 ± 7.55^AB^	46.09 ± 7.25^B^
LDL-C (mg/dl)	19.15 ± 2.96^A^	16.75 ± 3.27^AB^	15.22 ± 4.40^B^
Triglyceride (mg/dl)	146.18 ± 33.76^B^	262.40 ± 110.75^A^	246.70 ± 74.18^A^
Glucose (mg/dl)	230.40 ± 41.31^AB^	236.50 ± 35.53^A^	200.36 ± 37.09^B^
Leptin (pg/ml)	433.88 ± 117.15^B^	575.68 ± 149.86^A^	538.08 ± 158.12^AB^
AST (U/L)	98.50 ± 33.78^AB^	111.00 ± 13.39^A^	84.45 ± 13.77^B^
ALT (U/L)	38.00 ± 5.25^B^	65.00 ± 8.16^A^	62.70 ± 4.35^A^

Each value provided is the mean ± standard deviation. Values of each group with the same letters for each serological factor were not significantly different (*P *< 0.05).

**Table 6:** Serum levels of α-amylase and lipase

	SD	HFD	HFD-LAB
α-amylase (U/L)	182.36 ± 10.73	172.33 ± 12.51	191.57 ± 16.16
Lipase (U/L)	91.10 ± 21.38^B^	224.25 ± 33.21^A^	216.52 ± 22.52^A^

Each value provided is the mean ± standard deviation. Values of each group with the same letters for each serological factor were not significantly different (*P *< 0.05).

### Fecal total LAB counts

**Figure 2:**
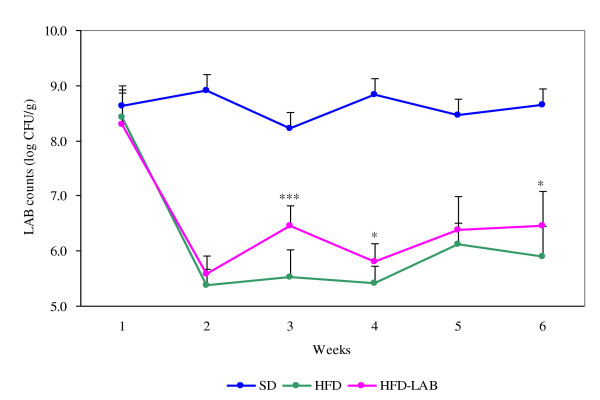
**Changes of total LAB counts during experimental periods**. Data are presented as means and standard deviation. *P < 0.05 and ***P < 0.001 significantly different compared with HFD group.

Fecal total LAB counts are shown in Figure 2. The fecal total LAB counts were clearly lower in the HFD-fed groups than in the SD group over the experimental period. However, after treatment with LAB, the counts in the HFD-LAB group were significantly increased compared with those in the HFD group.

### Harmful enzyme activities of intestinal microflora

**Figure 3:**
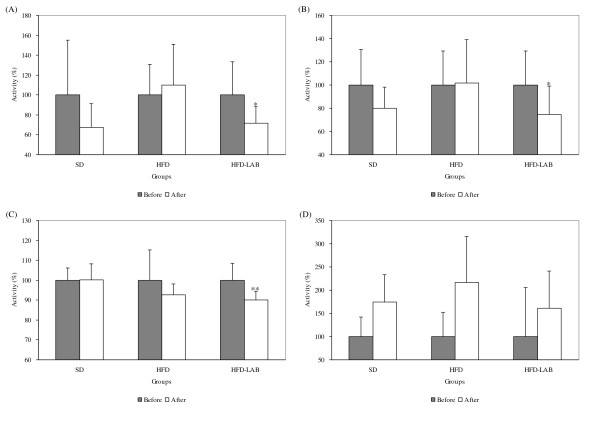
**Inhibitory effects of LAB on fecal harmful enzymes in each experimental group**. (A) β-glucosidase activity, (B) β-glucuronidase activity, (C) Tryptophanase activity, and (D) Urease activity. *P < 0.05 and **P < 0.01 significantly different compared with before LAB treatment.

As shown in Figure 3, the harmful enzyme activities of intestinal microflora in the HFD-LAB group were obviously decreased compared with those in the other groups. After LAB treatment, β-glucosidase, β-glucuronidase, and tryptophanase activities were decreased by 28%, 26% and 10%, respectively. In addition, there was a statistically significant decrease in β-glucosidase (*P *= 0.023), β-glucuronidase (*P *= 0.050), and tryptophanase (*P *= 0.002) activities. However, urease activities were increased.

## Discussion

Many studies have reported antiobesity effects of some bacterial strains such as *Lactobacillus *spp. and *Bifidobacterium *spp. [17-19,8]. In the present study, we observed that feeding of a high fat diet for 5 weeks produced significant increases in body weight, and administration of LAB reduced body weight gain and fat weight. However, there was no significant difference in spleen, kidney, and liver weights.

Our results demonstrated that the TC, HDL-C, and LDL-C levels in serum were decreased in the HFD-LAB group. The reduction of TC or LDL in serum is reported to lower the risk of coronary heart disease [20]. Recent studies showed that probiotics, including *Bifidobacterium longum *[6], *Lactobacillus acidophilus *[21], had hypocholesteremic effects in both rat and human. The mechanisms involved may be as follows [22-27]: (1) fermentation products of lactic acid bacteria inhibit cholesterol synthesis enzymes and thus reduce cholesterol production; (2) the bacteria facilitate the elimination of cholesterol in feces; (3) the bacteria inhibit the absorption of cholesterol back into the body by binding with cholesterol; (4) the bacteria interfere with the recycling of bile salt (a metabolic product of cholesterol) and facilitate its elimination, which raises the demand for bile salt made from cholesterol and thus results in body cholesterol consumption; and (5) the assimilation of lactic acid. In addition, we found that levels of triglyceride, glucose, AST, and ALT in serum were reduced in HFD-LAB group. Similar results have been observed with some specific probiotics strains [19,8,17].

Leptin has been identified as an antiobesity hormone that regulates body weight by controlling food intake and energy expenditure via the hypothalamic-pituitary-gonadal axis [28-30]. It appears that leptin may be an important factor in obesity management. Leptin is a 16 KDa secreted protein produced by the ob gene. Adipose tissue produces leptin and releases it into the bloodstream. As fat deposits grow, blood leptin levels tend to increase. Thus, leptin levels are closely related to the percentage of body fat; markedly higher serum leptin levels have been found in obese individuals compared with non-obese individuals [31,32]. In contrast, leptin levels are severely reduced in underweight individuals compared with normal weight individuals [33,34]. In the present study, leptin levels were higher in the HFD-fed groups than in the SD group, but were lower in the HFD-LAB group than in the HFD group. Similar effects have been observed in other studies using mouse [17] and human [32]. The results of these studies suggest that reductions in fat mass and body weight are associated with a reduction in leptin.

α-Amylase, a digestive enzyme secreted from the pancreas and salivary gland, is involved in important biological processes such as digestion of carbohydrates. Many crude drugs inhibit α-amylase activity [35]. Natural α-amylase inhibitors are beneficial in reducing post-prandial hyperglycemia by slowing down the digestion of carbohydrates and, consequently, the absorption of glucose. Reducing post-prandial hyperglycemia prevent glucose uptake into adipose tissue to inhibit synthesis and accumulation of triacylglycerol [36]. On the other hand, it is well known that dietary lipid is not directly absorbed from the intestine unless it has been subjected to the action of pancreatic lipase. The two main products formed by the hydrolysis of pancreatic lipase are fatty acid and 2-monoacylglycerol. Inhibition of these digestive enzymes is therefore beneficial in the treatment of obesity [37]. Our results showed that lipase levels were slightly decreased in the HFD-LAB group, whereas α-amylase levels were increased. That is, LAB had an inhibitory effect on lipase.

In our study, fecal LAB counts were obviously decreased in the HFD-fed groups compared with those in the SD group. Similarly, Cani et al. [38] demonstrated that a high fat diet changed the intestinal microbiota composition; in particular, the number of *Bifidobacterium *spp. was reduced. Kalliomaki et al. [39] also observed that fecal *Bifidobacterium *spp. counts were higher in children who remained at normal weight at the age of seven, while this was not the case in overweight children. After treatment with LAB, we found that the LAB counts in the HFD-LAB group were significantly increased compared with those in the HFD group. This result means that LAB survive passage through the upper-gastrointestinal tract after oral feeding, and ingested LAB affect the intestinal environment to favor LAB colonization [40].

Several studies have shown that these specific bacteria reduce the intestinal endotoxin levels and improve mucosal barrier function [41-43]. Furthermore, LAB have anti-tumor effects and block harmful intestinal enzyme activities, a recognized risk factor for colon cancer [44,45]. The results of the present study showed the harmful enzyme activities of intestinal microflora in the HFD-LAB group were clearly decreased compared with those in the other groups; in particular, the activities of β-glucosidase, β-glucuronidase, and tryptophanase were significantly decreased.

## Conclusion

In conclusion, we suggest that the LAB supplement (*B. pseudocatenulatum *SPM 1204, *B. longum *SPM 1205, *B. longum *SPM 1207) used in this study may have beneficial antiobesity effects. Further clinical trials to confirm these effects should therefore be conducted.

## Competing interests

The authors declare that they have no competing interests.

## Authors' contributions

This study was conceived by NJH and designed by NJH and KJK. NJH and KJK were responsible for obtaining funding and sample collection. The animal experiments, blood serum levels, fecal total LAB counts and harmful enzyme activities test were done by HMA, SYP, DKL, JRK, MKC, SWL and LHT. HMA performed data analysis and wrote the draft of the manuscript. All authors read and approved the final manuscript.
